# Translation, cross-cultural adaptation, validation, and diagnostic properties of the Arabic version of self-diagnosis questionnaire for Benign Paroxysmal Positional Vertigo

**DOI:** 10.3389/fneur.2026.1834087

**Published:** 2026-05-13

**Authors:** Ahmad A. Alharbi, Mohammad B. Alkhodair, Abdulaziz A. Albalwi, Hamad S. Al Amer, Akram M. Abdelrahman, Faisal M. Alzuhair, Nawaf N. Alsulami, Abrar H. Alhazmi

**Affiliations:** 1 Department of Health Rehabilitation Sciences, Faculty of Applied Medical Sciences, University of Tabuk, Tabuk, Saudi Arabia; 2 Audiology and Speech Pathology Department, King Salman Armed Forces Hospital, Tabuk, Saudi Arabia; 3 Department of Languages and Translation, Faculty of Education and Arts, University of Tabuk, Tabuk, Saudi Arabia; 4 English Language Center, University of Tabuk, Tabuk, Saudi Arabia

**Keywords:** Arabic, Benign Paroxysmal Positional Vertigo, diagnostic accuracy, reliability, validity

## Abstract

Benign Paroxysmal Positional Vertigo (BPPV) is a common peripheral vestibular disorder, yet no validated Arabic BPPV-specific questionnaire is currently available. To translate and culturally adapt the self-diagnosis BPPV questionnaire into Arabic and to evaluate its psychometric and diagnostic properties. Following cross-cultural adaptation in accordance with established guidelines, 88 participants completed the Arabic version of the questionnaire. Psychometric and diagnostic properties were evaluated. Content validity was excellent (item-level indices ranging from 0.94 to 1.00 for both clarity and relevance, and a scale-level index of 0.98). Convergent validity demonstrated moderate to substantial agreement with positional test results (Cohen's κ values reaching 0.69). Known-groups validity showed fair to excellent discriminative ability (area under the curve values up to 0.84). Internal consistency was moderate for the screening questions (Cronbach's α = 0.720) but lower for subtype determination questions (Cronbach's α = 0.532). The screening component correctly classified (81.8%) participants, with sensitivity, specificity, PPV, NPV, PLR, and NLR of 87.2%, 75.6%, 80.4%, 83.8%, 3.58, and 0.17, respectively. Agreement for the affected ear and BPPV subtype was limited, with many undetermined cases. The Arabic self-diagnosis BPPV questionnaire demonstrated strong content validity, acceptable construct validity, and good diagnostic performance for screening BPPV.

## 1 Introduction

Benign Paroxysmal Positional Vertigo (BPPV) is the most common peripheral vestibular disorder (1, 2) characterized by brief, recurrent episodes of vertigo and nystagmus triggered by changes in head position due to displaced otoconia within the semicircular canals (3, 4). Although BPPV is benign and treatable, its sudden and unpredictable attacks frequently impair mobility, restrict participation in daily activities, and contribute to anxiety and fear of falling (5–7), collectively diminishing quality of life (8, 9).

Globally, BPPV accounts for approximately 17%−42% of dizziness or vertigo cases seen in specialized clinics (2, 10, 11). The condition is more common among women and older adults (1, 2). Dizziness is a common issue in Saudi Arabia, with a recent study reporting its prevalence to be approximately 43% (12). A retrospective study conducted in Saudi Arabia at a tertiary-care center found that 39.5% of vertigo patients were diagnosed with BPPV, making it the single most common cause of vertigo in that population, followed by Meniere's disease (27.4%) (13). The same study reported that peripheral vestibular disorders accounted for 73.4% of all vertigo diagnoses in their cohort (13).

BPPV has a high tendency to recur, with reported annual recurrence rates ranging from 15 to 18% (3, 14, 15). If recurrent episodes consistently involved the same BPPV subtype and affected semicircular canal as the initial episode, patients could be taught to perform canalith repositioning procedures (CRPs) independently and apply them when symptoms reappear. However, recurrent BPPV does not reliably involve the same canal or subtype as the previous episode (16). This inconsistency restricts the routine recommendation of repeating previously effective maneuvers. In this context, Kim et al. developed a questionnaire intended to support patient self-identification of BPPV that would allow self-administration of an appropriate CRPs, particularly in cases of recurrent BPPV (17).

Patient-reported outcome measures (PROMs) are integral to vestibular assessment, as objective vestibular tests often correlate poorly with patients' subjective symptoms and functional limitations (18–20). Disease-specific PROMs provide greater sensitivity than generic measures (21, 22); enabling clinicians to quantify dizziness severity, functional impairment, and psychosocial impact—key elements for appropriate diagnosis, treatment planning, and outcome monitoring (23, 24). Although several validated vestibular PROMs exist internationally (18, 19), to our knowledge no validated Arabic BPPV-specific questionnaire is currently available.

Arabic is the official language in 22 countries with 456 million Arabic speakers around the world (25). Using non-validated translations risks semantic inaccuracies and undermines measurement reliability (26, 27), limiting both clinical quality and research comparability across Arabic-speaking populations. Additionally, self-diagnosis questionnaires provide a cost-effective and easily administered approach, enable assessment of large populations, and may facilitate and support disease self-management. Therefore, this study aimed to translate and culturally adapt a BPPV-specific questionnaire into Arabic and to evaluate its psychometric and diagnostic properties. By doing so, we intend to provide a valid instrument for assessing BPPV symptoms and impact in Arabic-speaking patients, facilitating better clinical care and enabling robust regional research on BPPV.

## 2 Material and methods

### 2.1 Study design and setting

This cross-sectional methodological study was conducted to translate, culturally adapt, and validate the Questionnaire-based Diagnosis of Benign Paroxysmal Positional Vertigo (BPPV-Q) for Arabic-speaking populations. The study was performed at the audiology clinics of King Salman Armed Forces Hospital (KSAFH), a tertiary-care facility in Tabuk, northwestern region of the Kingdom of Saudi Arabia, between June 2025 and November 2025. The study protocol was reviewed and approved by the Institutional Review Board at KSAFH (approval number: KSAFH-2025-645). All participants provided written informed consent prior to enrollment.

### 2.2 Translation and cross-cultural adaptation

Necessary permission to translate and validate the BPPV-Q was obtained from the original instrument developers (17). This study followed guidelines for process of cross-cultural adaptation of self-report measures proposed by Beaton et al. (28). Phase I comprised the translation and cross-cultural adaptation process including five steps. Phase II involved psychometric validation, including assessment of construct validity, diagnostic accuracy against positional tests, and internal consistency.

Phase I: Translation and cross-cultural adaptation process.

Step 1: Forward translation.

Bilingual translators who were native Arabic speakers with fluency in English performed two independent forward translations from English to Arabic. Translator 1 was a clinical audiologist with 10 years of experience in vestibular disorders. Translator 2 was a lecturer in the Languages and Translation Department at the University of Tabuk with neither a medical background nor awareness of the BPPV-Q concept.

Step 2: Translation synthesis.

The researcher held a meeting with the two translators to synthesize the two forward translations. They achieved an agreement and formed a single questionnaire (synthesized Arabic questionnaire).

Step 3: Back-translation.

Two translators worked independently to produce English versions of the synthesized Arabic questionnaire. Both back-translators held doctoral degrees in linguistics and translation studies and had no prior exposure to the original BPPV-Q or knowledge of BPPV; additionally, they were instructed not to search for the questionnaire.

Step 4: Expert committee review.

An expert committee, consisting of a methodologist, a linguistic expert, a physical therapist, an audiologist, and professional translators, undertook a comprehensive review of all versions to resolve discrepancies and ensure semantic, idiomatic, experiential, and conceptual equivalence between the original and Arabic versions. Necessary changes were made to ensure clarity and suitability for the general Arabic public and subsequently presented the pre-final Arabic version of BPPV-Q.

Step 5: Pretesting.

A panel 20-health professional (five audiologists, five otorhinolaryngologists, five physical therapists, and five neurologists) evaluated the pre-final Arabic version for content validity. They independently assessed the clarity and relevance of each item using a 4-point ordinal scale (1 = unclear/irrelevant, 2 = somewhat clear/somewhat relevant, 3 = clear/relevant, and 4 = very clear/very relevant). Subsequently, pilot tested on 20 participants to assess clarity, comprehensibility, and cultural appropriateness. There were no significant challenges reported. The aforementioned procedures culminated in the ultimate Arabic version of BPPV-Q (Arabic BPPV-Q), which was employed in the psychometric assessment. The panel of 20 health professionals was not included for psychometric validation.

Phase II: Psychometric validation.

### 2.3 Participants and sampling

Participants were recruited consecutively from patients presenting to the audiology clinics at KSAFH with dizziness as the chief complaint. Eligibility criteria were established a priori and applied systematically. Participants who aged ≥18 years, presenting complaint of dizziness or vertigo, spoke Arabic as the native language, abled to provide informed, and willing to complete the study procedures included in this study. However; exclusion criteria included history of diagnosed neurological disorders (e.g., stroke, multiple sclerosis, Parkinson's disease), history of psychiatric disorders requiring ongoing treatment, severe visual impairment preventing questionnaire completion, cervical spine pathology requiring ongoing treatment, Illiteracy or inability to comprehend written Arabic, cognitive impairment (as assessed by clinical judgment or documented diagnosis). A control group of healthy volunteers with no history of dizziness, vertigo, or vestibular disorders was recruited from hospital staff and community members to assess discriminant validity. Control participants met the same age and language criteria and had no exclusion criteria.

Sample size was determined based on established guidelines for psychometric validation studies. For construct validity assessment through factor analysis, a minimum of 5–10 participants per questionnaire item is recommended (29). With six items in the BPPV-Q, a minimum of 30–60 participants would be required. For diagnostic accuracy studies, sample size should provide adequate precision for sensitivity and specificity estimates; a sample of 40 cases with BPPV provides 95% confidence intervals of approximately ±15% around point estimates of sensitivity and specificity (30). The inclusion of 40 participants with dizziness symptoms (anticipated to include both BPPV-positive and BPPV-negative cases) and 40 healthy controls (total *n* = 80) exceeds minimum requirements and provides adequate statistical power for planned analyses.

### 2.4 Procedures

All eligible participants completed a standardized assessment protocol administered by trained research personnel. The assessment comprised:

- Demographic and clinical data collection: age, sex, duration of symptoms, previous vestibular diagnoses, and relevant medical history were recorded using a structured case report form.
- Arabic BPPV-Q administration: participants completed the questionnaire independently in a quiet, well-lit clinic room. A trained researcher was available to clarify instructions if needed but did not assist with item responses. Questionnaires were scored according to the original algorithm: questions 1–3 determine BPPV likelihood (all three “yes” responses indicate probable BPPV); Questions 4–6 provide canal and subtype localization.
- Gold-standard diagnostic testing: all participants underwent standardized positional testing performed by a certified audiologist with specialized training in vestibular assessment after completing the Arabic BPPV-Q. The testing protocol included the Dix-Hallpike maneuver for posterior and anterior semicircular canal and the supine roll test for horizontal semicircular canal. All tests were performed with videonystagmography (VNG) recording to objectively document nystagmus characteristics (direction, duration, intensity). The audiologist was blinded to the participant's BPPV-Q responses. BPPV diagnosis and subtype classification were determined based on the presence and characteristics of positional nystagmus according to established diagnostic criteria (1).

### 2.5 Questionnaire description

The BPPV-Q is a self-administered diagnostic questionnaire originally developed and validated in Korean and English by Kim et al. (17). The instrument consists of six structured questions designed to identify the presence of BPPV and determine the affected semicircular canal and subtype. Questions 1–3 assess cardinal BPPV characteristics (episodic rotational vertigo, positional triggers, and duration < 1 minute) to differentiate BPPV from other vestibular disorders. Questions 4–6 provide diagnostic localization: question 4 identifies the affected canal plane (vertical vs. horizontal) based on specific positional triggers (lying down/getting up vs. turning in bed); Question 5 determines laterality (right vs. left); and Question 6 distinguishes canalolithiasis vs. cupulolithiasis based on symptom duration. Each question employs a dichotomous (yes/no) or categorical response format. The original validation study reported high sensitivity (87.0%) and specificity (89.8%) for BPPV diagnosis, with good agreement (κ = 0.74) with clinical diagnostic testing (17).

### 2.6 Data analysis

The statistical analysis comprised two main components: psychometric evaluation and assessment of the diagnostic properties of the translated questionnaire. Psychometric analyses were conducted to examine validity and reliability. Diagnostic property analyses were performed to evaluate the ability of the questionnaire to identify BPPV relative to the reference standard provided by positional test. All psychometric and diagnostic property outcomes were evaluated against *a priori* hypotheses (Table 1), which were formulated based on findings from previous studies of the self-diagnosis BPPV questionnaire (18) and established recommendations for psychometric properties reported in published methodological guidelines (31).

**Table 1:** *A priori* hypotheses for the psychometric and diagnostic properties evaluation of the Arabic self-diagnosis BPPV questionnaire.

Reliability		
Internal consistency	Cronbach's α = 0.70–0.95 [31]	
Validity		
**Content validity**	I-CVI ≥ 0.78 [33] S-CVI/Ave ≥ 0.90 [33] S-CVI/UA ≥ 0.80 [33]	
Construct validity	Test	Hypothesis
Convergent validity	Dix-Hallpike	Substantial agreement (κ = 0.610–0.740) [17]
**Known-groups validity**		AUC > 0.70 [31]
Diagnostic performance		
**Accuracy**	56.1–88.9 [17, 37, 38]	
**Sensitivity**	77.6–95.2 [17, 37, 38]	
**Specificity**	37.5–89.8 [17, 37, 38]	
**PPV**	42.0–80.0 [17, 37, 38]	
**NPV**	84.5–94.8 [17, 37, 38]	
**PLR**	1.52–8.57 [17, 37, 38]	
**NLR**	0.12–0.38 [17, 37, 38]	

BPPV, Benign Paroxysmal Positional Vertigo; I-CVI, Item-Level Content Validity Index; S-CVI/Ave, Average Scale-Level Content Validity Index; S-CVI/UA, universal agreement; κ, Cohen's kappa coefficient; AUC, area under the curve; PPV, positive predictive value; NPV, negative predictive value; PLR, positive likelihood ratio; NLR, negative likelihood ratio.

### 2.7 Validity

#### 2.7.1 Content validity

Content validity of the Arabic questionnaire was evaluated by calculating item-level content validity indices (I-CVI), defined as the proportion of experts rating an item as clear and relevant (scores of 3 or 4 on a 4-point scale), scale-level content validity that was assessed using the average of the I-CVI values (S-CVI/Ave), and the universal agreement index (S-CVI/UA), defined as the proportion of items with an I-CVI of 1.00 (32). Content validity indices were interpreted according to established criteria, with higher values indicating stronger content validity (33).

#### 2.7.2 Construct validity

*Convergent validity:* convergent validity was assessed by examining the agreement between the questionnaire-based classification and the reference standard classification. Agreement was quantified using Cohen's kappa coefficient (κ), which was interpreted as follows: values of 0.01–0.20 indicated slight agreement; 0.21–0.40 fair agreement; 0.41–0.60 moderate agreement; 0.61–0.80 substantial agreement; and 0.81–0.99 almost perfect agreement (34).

#### 2.7.3 Known-groups validity

Known-groups validity was evaluated by examining the ability of the Arabic questionnaire to discriminate between predefined groups based on the reference standard. Receiver operating characteristic (ROC) curve analysis was performed, and the area under the ROC curve (AUC) was calculated as a measure of discriminative ability. AUC values were interpreted as follows: values ≥ 0.90 were considered excellent; 0.80–0.89 considerable; 0.70–0.79 fair; 0.60–0.69 poor; and 0.50–0.59 indicating failure to discriminate between groups (35).

### 2.8 Reliability

Reliability of the translated questionnaire was assessed by examining internal consistency using Cronbach's alpha (α). Cronbach's α values were interpreted as follows: values < 0.50 indicated poor reliability; 0.50–0.75 moderate reliability; 0.75–0.90 good reliability; and values > 0.90 excellent reliability (36).

### 2.9 Diagnostic property

The diagnostic properties of the Arabic questionnaire were evaluated using the results of the Dix–Hallpike and positional tests as the reference standard. Diagnostic performance metrics included accuracy, sensitivity, specificity, positive predictive value (PPV), negative predictive value (NPV), positive likelihood ratio (PLR), and negative likelihood ratio (NLR). These metrics were calculated for individual questionnaire items as well as for the overall questionnaire classification.

### 2.10 Statistical tests and software

All statistical analyses were performed using Microsoft Excel (Microsoft Corp., Redmond, WA, USA) and IBM SPSS Statistics for Windows version 25.0 (IBM Corp., Armonk, NY, USA). Comparisons between groups were conducted using the Mann–Whitney *U*-test for continuous variables and the chi-square test for categorical variables with an alpha level of 0.05.

## 3 Result

### 3.1 Participant characteristics and reference standard findings

A total of 88 participants were included in the analysis. Based on the reference standard assessment using the Dix–Hallpike and positional tests, 47 participants (53.4%) were diagnosed with BPPV, while 41 participants (46.6%) had negative test results (Table 2). Participants with BPPV were significantly older than those without BPPV (mean ± SD: 52.4 ± 11.7 vs. 43.5 ± 15.2 years; *p* = 0.002). There was no significant difference in sex distribution between the BPPV-positive and BPPV-negative groups (male: 53.2 vs. 56.1%, *p* = 0.785). Among participants with confirmed BPPV, the affected ear was almost evenly distributed between the right (51.1%) and left (48.9%) sides, and all BPPV cases were classified as posterior canal involvement based on positional testing.

**Table 2:** Characteristics of the participants (*n* = 88).

Characteristic	BPPV + (*n* = 47)	BPPV - (*n* = 41)	*p*-value	Overall
Age mean ± SD (years)	52.4 ± 11.7	43.5 ± 15.2	0.002^*^	48.2 ± 14.1
Sex				
Male	25 (53.2)	23 (56.1)	0.785	48 (54.5)
Female	22 (46.8)	18 (43.9)		40 (45.5)
Affected ear				
Right	24 (51.1)	–		
Left	23 (48.9)	–		
BPPV type				
Posterior	47 (100.0)	–		

Values are presented as numbers (percentages) unless otherwise specified; Mann–Whitney U or chi-square tests were used for group comparisons. SD, standard deviation; BPPV, benign paroxysmal positional vertigo. ^*^Significant at α = 0.05.

### 3.2 Validity

#### 3.2.1 Content validity

The results of the content validity assessment are presented in Table 3. All six items of the Arabic questionnaire demonstrated acceptable item-level content validity for both clarity and relevance, with no items exhibiting an I-CVI < 0.70. For both components, I-CVI values ranged from 0.95 to 1.00. The S-CVI/Ave was 0.98 for both clarity and relevance, indicating excellent overall content validity of the questionnaire. S-CVI/UA indicis were 0.67 for both clarity and relevance. Except for S-CVI/UA, all these values are consistent with the predefined hypotheses for content validity of the questionnaire.

**Table 3:** Results of content validity assessment of the Arabic self-diagnosis BPPV questionnaire (*n* = 20).

Variable	Clarity component	Relevance component
Number of Items with I-CVI ≥ 0.70	6	6
Number of Items with I-CVI < 0.70	0	0
Minimum–Maximum I-CVI	0.95–1.00	0.95–1.00
S-CVI/Ave	0.98	0.98
S-CVI/UA	0.67	0.67

BPPV, Benign Paroxysmal Positional Vertigo; I-CVI, Item-Level Content Validity Index; S-CVI/Ave, Average Scale-Level Content Validity Index; S-CVI/UA, universal agreement.

#### 3.2.2 Construct validity

*Convergent validity:* Convergent validity results are summarized in Table 4. Agreement between questionnaire-based classifications derived from the screening questions (questions 1–3) and the reference standard varied across individual items and item combinations. For individual items, κ values ranged from 0.34 (95% CI: 0.16–0.52) for question 3 to 0.56 (95% CI: 0.39–0.72) for question 1. Combining questions improved agreement, with κ values of 0.61 (95% CI: 0.43–0.78) for questions 1 and 2, and 0.63 (95% CI: 0.47–0.79) for the complete screening component of the questionnaire (questions 1–3). The overall questionnaire classification showed the highest level of agreement, with a κ value of 0.69 (95% CI: 0.49–0.88). Agreement estimates for combined item sets and the overall questionnaire classification were consistent with the predefined *a priori* hypotheses presented in Table 1.

**Table 4:** Results of construct validity assessment of the Arabic self-diagnosis BPPV questionnaire.

Question	*n*	Convergent validity	Known-Groups validity	
		κ **(95% CI)**	AUC (95% CI)	**SE**
1	88	0.56 (0.39–0.72)	0.77 (0.67–0.88)	0.05
2	88	0.53 (0.36–0.71)	0.76 (0.65–0.87)	0.05
1 + 2	88	0.61 (0.43–0.78)	0.80 (0.70–0.90)	0.05
3	88	0.34 (0.16–0.52)	0.66 (0.55–0.78)	0.06
1 + 2 + 3	88	0.63 (0.47–0.79)	0.81 (0.72–0.91)	0.05
5	41	0.32 (0.02–0.61)	0.66 (0.50–0.82)	0.07
Overall	70	0.69 (0.49–0.88)	0.84 (0.74–0.94)	0.05

Because horizontal canal BPPV was not identified by positional testing, canal-specific validity assessment related to question 4 and 6 could not be estimated. BPPV, benign paroxysmal positional vertigo; κ, Cohen's kappa coefficient; AUC, area under the curve; SE, standard error.

#### 3.2.3 Known-groups validity

Known-groups validity findings are also presented in Table 4. The ability of the screening questions (questions 1–3) to discriminate between predefined groups based on Dix–Hallpike test results demonstrated fair to excellent discriminative performance across individual items and item combinations. AUC values ranged from 0.66 (95% CI: 0.55–0.78) for question 3 to 0.77 (95% CI: 0.67–0.88) for question 1. Improved discrimination was observed when questionnaire items were combined, with AUC values of 0.80 (95% CI: 0.70–0.90) for questions 1 and 2, and 0.81 (95% CI: 0.72–0.91) for all screening questions combined. The overall questionnaire classification demonstrated the highest discriminative ability, with an AUC of 0.84 (95% CI: 0.74–0.94). With the exception of question 3, these AUC values were consistent with the *a priori* hypotheses regarding known-groups validity of the questionnaire.

### 3.3 Reliability

The screening questions (questions 1–3) of the Arabic questionnaire demonstrated moderate internal consistency, with a Cronbach's α of 0.720, meeting the predefined *a priori* hypothesis. However, questions assessing determination of the affected ear and BPPV subtypes (questions 4–6) yielded a Cronbach's α of 0.532, which was below our hypothesized internal consistency value.

### 3.4 Diagnostic property results

**Figure 1:**
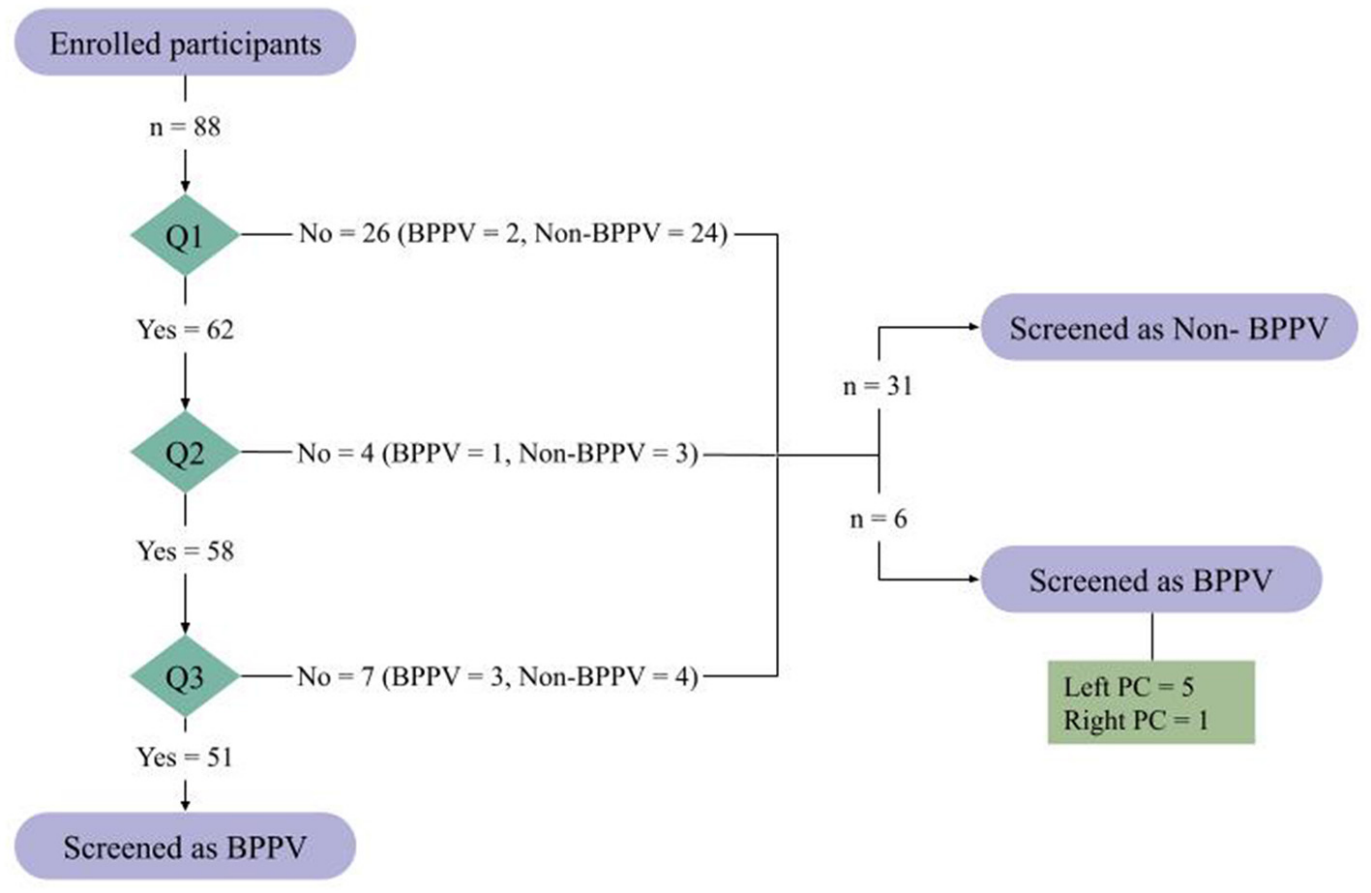
Participant classification based on questionnaire responses.

*Screening performance for BPPV (Questions 1–3):* Of the 88 participants included in the study, 37 answered “no” to at least one of questions 1 through 3 and were therefore classified by the questionnaire as having conditions other than BPPV (Figure 1). Among these participants, 6 (16.2%) were subsequently found to have BPPV based on the Dix–Hallpike reference standard, representing false-negative classifications. The remaining 31 participants (83.8%) were confirmed to be non-BPPV on positional testing.

**Figure 2:**
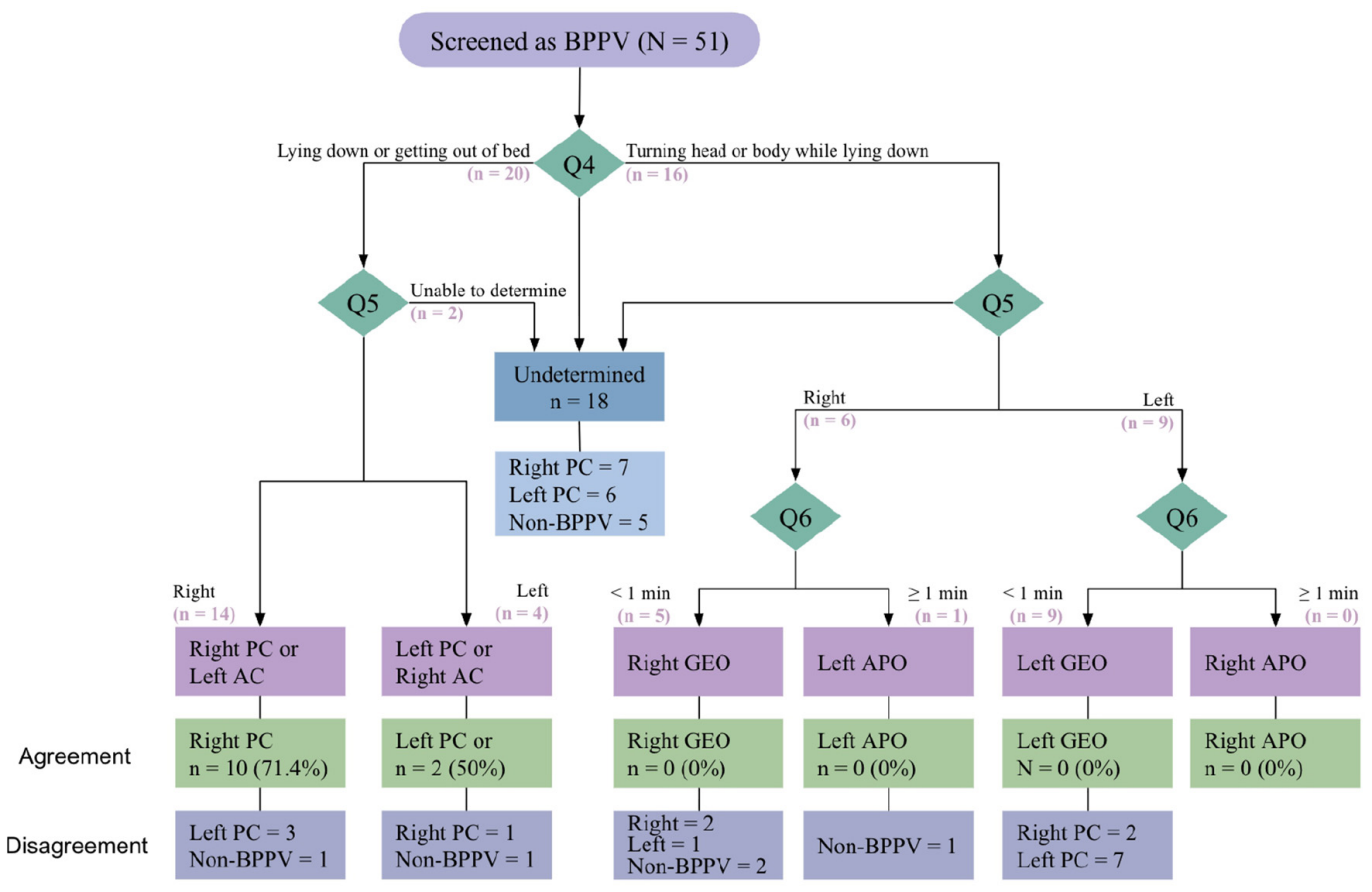
Screening outcomes based on the translated questionnaire.

In contrast, 51 participants were screened as having BPPV by the translated questionnaire (Figure 2). Of these, 41 (80.4%) were confirmed to have BPPV by the reference standard, with all cases identified as PC-BPPV. Conversely, 10 (19.6%) did not demonstrate positional nystagmus consistent with BPPV during Dix–Hallpike testing and were therefore classified as non-BPPV.

As shown in Table 5, the diagnostic accuracy of the screening component improved progressively with the sequential combination of individual questions. Accuracy increased when questions 1 and 2 were combined and further improved when all three screening questions (questions 1–3) were used together. Overall, the screening questions correctly classified 72 of 88 participants with respect to the presence or absence of BPPV. The combined screening demonstrated a sensitivity of 87.2%, a specificity of 75.6%, and an overall diagnostic accuracy of 81.8%. The PPV and NPV values were 80.4 and 83.8%, respectively, with corresponding likelihood ratios of 3.58 for a positive result and 0.17 for a negative result.

**Table 5:** Diagnostic properties of the Arabic self-diagnosis BPPV questionnaire.

Question	*N*	Accuracy (95% CI)	Sensitivity (95% CI)	Specificity (95% CI)	PPV (95% CI)	NPV (95% CI)	PLR (95% CI)	NLR (95% CI)
1	88	78.4 (68.1–86.6)	95.7 (85.0–99.2)	58.5 (42.3–73.9)	72.6 (59.9–82.9)	92.3 (74.6–99.0)	2.31 (1.51–3.54)	0.07 (0.02–0.26)
2	88	77.3 (67.5–85.5)	95.7 (85.0–99.2)	56.1 (39.6–71.6)	71.4 (58.8–81.9)	92.0 (74.1–98.9)	2.18 (1.43–3.34)	0.08 (0.02–0.32)
1 + 2	88	80.7 (71.0–88.2)	93.6 (82.8–98.5)	65.9 (49.4–79.7)	75.9 (62.9–85.7)	90.0 (73.4–97.9)	2.74 (1.65–4.56)	0.10 (0.04–0.27)
3	88	68.2 (57.6–77.7)	93.6 (82.8–98.5)	39.0 (24.1–55.6)	63.8 (51.5–74.6)	84.2 (60.1–96.4)	1.54 (1.11–2.13)	0.16 (0.05–0.49)
1 + 2 + 3	88	81.8 (72.4–89.1)	87.2 (74.2–94.9)	75.6 (60.2–87.1)	80.4 (67.6–89.7)	83.8 (68.9–93.3)	3.58 (2.04–6.28)	0.17 (0.07–0.39)
4	41	41.5 (27.7–56.7)	–					
5	41	65.8 (49.4–79.9)	68.4 (43.5–87.4)	63.6 (40.7–82.8)	61.9 (38.4–81.9)	70.0 (45.7–88.1)	1.88 (1.00–3.54)	0.50 (0.24–1.03)
Overall	70	84.3 (73.3–92.0)	82.4 (66.7–92.7)	86.1 (70.8–95.3)	84.8 (68.9–94.4)	83.8 (68.9–93.4)	5.93 (2.49–14.11)	0.20 (0.09–0.43)

Because horizontal canal BPPV was not identified by positional testing, canal-specific diagnostic performance metrics, including those related to question 6, could not be fully estimated. BPPV, Benign Paroxysmal Positional Vertigo; PPV, positive predictive value; NPV, negative predictive value; PLR, positive likelihood ratio; NLR, negative likelihood ratio.

Taken together, the diagnostic performance indices for accuracy, sensitivity, specificity, PPV, NPV, PLR, and NLR were comparable to the predefined hypothesis ranges. Further, after excluding participants with incomplete responses and considering the overall questionnaire classification, diagnostic performance remained robust. In this restricted sample (*n* = 70), the Arabic questionnaire demonstrated an accuracy of 84.3% (59/70), sensitivity of 82.4%, specificity of 86.1%, PPV of 84.8%, and NPV of 83.8%, with corresponding likelihood ratios of 5.93 for a positive result and 0.20 for a negative result. All these indices met or exceeded the *a priori* hypotheses for diagnostic performance.

### 3.5 Determination of the affected ear and BPPV subtype (questions 4–6)

Among the 51 participants who were identified as positive for BPPV based the screening questions, agreement between questionnaire-based classification and the Dix–Hallpike reference standard with respect to the affected canal and BPPV subtype was observed in 12 participants (23.5%), whereas 21 participants (41.2%) showed disagreement (Figure 2). The remaining 18 participants (35.3%) were classified as undetermined, primarily due to endorsement of multiple positional triggers, head direction, or incomplete responses to questions 4 through 6. Of these undetermined cases, five participants (27.8%) did not demonstrate nystagmus during positional testing.

*Question 4: Positional triggers:* Among the 41 participants with a final diagnosis of PC-BPPV based on the reference standard, 17 (41.5%) reported experiencing more severe vertigo when lying down or getting out of bed. Although no participants were diagnosed with HC-BPPV in this subgroup, 13 participants (31.7%) reported greater vertigo when turning the head or body while lying down. The remaining 11 participants (26.8%) reported that both positional triggers provoked their vertigo and were therefore classified as undetermined. Accordingly, the diagnostic accuracy of question 4 for identifying the positional trigger consistent with PC-BPPV was 41.5% (Table 5). This accuracy value was below the predefined hypothesis range for diagnostic accuracy.

*Question 5: Direction of maximal symptoms:* Question 5 correctly identified the affected ear in 27 of 41 participants (65.8%) with confirmed BPPV (Figure 2). In contrast, 14 participants (34.2%) demonstrated discordance between the questionnaire-based side determination and the Dix–Hallpike reference standard. Overall, question 5 showed relatively lower diagnostic accuracy for determining the affected ear in this sample (Table 5); however, the observed accuracy remained consistent with the predefined hypothesis threshold.

*Question 6: Duration of vertigo episodes:* Question 6 is applicable to participants with responses consistent with horizontal canal involvement and was designed to distinguish between geotropic and apogeotropic subtypes based on symptom duration. However, calculation of diagnostic properties for this question was not possible, as no cases of HC-BPPV were identified in this sample based on positional testing.

### 3.6 Comparison between accurate and inaccurate classifications

A comparison of demographic and clinical characteristics between participants with accurate and inaccurate questionnaire-based classifications among those with a final diagnosis of BPPV is presented in Table 6. Of the 47 participants with confirmed BPPV, 12 were accurately classified by the Arabic questionnaire, while 35 were inaccurately classified. There were no significant differences between the accurate and inaccurate classification groups with respect to age (*p* = 0.714) or sex distribution (*p* = 0.797). However, the affected ear differed significantly between groups, with right-sided involvement being more frequent among accurately classified participants (*p* = 0.017).

**Table 6:** Comparative analysis between accurate and inaccurate classification groups among patients with a final diagnosis of BPPV (*n* = 47).

Characteristic	Accurate (*n* = 12)	Inaccurate (*n* = 35)	*p*-value
Age mean ± SD (years)	51.9 ± 9.7	52.5 ± 12.5	0.714
Sex			
Male	6 (50.0)	19 (54.3)	0.797
Female	6 (50.0)	16 (45.7)	
Affected ear			
Right	10 (83.3)	14 (40.0)	0.017^*^
Left	2 (16.7)	21 (60.0)	

Values are presented as numbers (percentages) unless otherwise specified; Mann–Whitney U or chi-square tests were used for group comparisons SD, standard deviation; BPPV, Benign Paroxysmal Positional Vertigo.

* Significant at α = 0.05.

## 4 Discussion

This study translated and cross-culturally adapted the self-diagnosis BPPV questionnaire into Arabic and evaluated its psychometric and diagnostic properties among Arabic-speaking patients presenting with vertigo. The Arabic version demonstrated excellent content validity, acceptable construct validity with moderate to substantial agreement with the Dix–Hallpike test, and good diagnostic performance for BPPV screening. Internal consistency was moderate for the screening component but lower for subtype determination questions. Known-groups validity showed fair to excellent discriminative ability. However, agreement between questionnaire-based and positional diagnoses for the affected ear and BPPV subtype was limited, with a substantial proportion of cases classified as undetermined.

The Arabic version achieved excellent content validity, with I-CVI ranging from 0.95 to 1.00 for both clarity and relevance, and S-CVI of 0.98. These values exceed the recommended thresholds of 0.78 for I-CVI and 0.90 for S-CVI (33), indicating that the expert panel unanimously agreed that the translated items were clear, relevant, and appropriate for the target population. This finding suggests that the cross-cultural adaptation process, conducted in accordance with Beaton et al. (28), guidelines, successfully maintained the conceptual equivalence of the original questionnaire while ensuring linguistic and cultural appropriateness for Arabic-speaking patients.

Convergent validity, assessed through agreement with the positional test as the reference standard, demonstrated moderate to substantial levels of concordance. The Cohen's κ value for overall classification agreement with positional testing was 0.69 in the Arabic version compared to 0.740 in the original study (17), both indicating substantial agreement (34). This indicates that the Arabic questionnaire performs comparably to clinical positional testing for identifying BPPV. This level of agreement is clinically meaningful and supports the questionnaire's utility as a screening tool in Arabic-speaking populations.

Known-groups validity, evaluated through the questionnaire's ability to discriminate between patients with and without BPPV, yielded AUC values up to 0.84. According to established interpretive criteria (35), AUC values between 0.80 and 0.90 indicate excellent discriminative ability. This finding confirms that the Arabic questionnaire can effectively differentiate between individuals with and without BPPV, supporting its construct validity within the studied population.

Internal consistency for the screening questions (Questions 1–3) was moderate (Cronbach's α = 0.720), meeting the acceptable threshold of 0.70 for group-level comparisons (31). This indicates adequate homogeneity among the items designed to screen for BPPV presence. However, internal consistency for the subtype determination questions (Questions 4–6) was lower (Cronbach's α = 0.532), falling below conventional acceptability standards. This lower value may reflect the heterogeneous nature of these items, which assess distinct constructs (canal involvement, affected side, and BPPV subtype) rather than a single underlying dimension.

The Arabic questionnaire demonstrated good diagnostic performance for BPPV screening. The sensitivity of 87.2% indicates that the questionnaire correctly identified the majority of patients with BPPV, minimizing false-negative results. The specificity of 75.6% suggests reasonable performance in correctly identifying patients without BPPV, though with a moderate false-positive rate. The PPV (80.4%) and NPV (83.8%) indicate that the questionnaire provides clinically useful information for ruling in and ruling out BPPV in the studied context. The PLR (3.58) and NLR (0.17) further support the questionnaire's utility, with the latter indicating that a negative result substantially reduces the probability of BPPV.

While sensitivity was nearly identical with original (17), specificity was approximately 14 percentage points lower in the Arabic version. The overall accuracy of the Arabic version (81.8%) was comparable to the original study's precision of 80.0% (17). These similarities in sensitivity suggest that the Arabic version maintains the original questionnaire's ability to correctly identify patients with BPPV. However, the lower specificity in the Arabic version indicates a higher false-positive rate, meaning that more patients without BPPV were incorrectly classified as having the condition. This difference may be attributable to several factors, including cultural differences in symptom interpretation and reporting, linguistic nuances in describing vestibular symptoms, or differences in patient populations. Additionally, the smaller sample size in the current study may have contributed to wider confidence intervals and greater variability in the specificity estimate.

The Arabic version's performance can also be compared with two Chinese adaptations of the same questionnaire reported by Wan et al. (37, 38). In the general Chinese population study, the questionnaire demonstrated sensitivity of 90.9%, specificity of 66.6%, and overall accuracy of 74.4% (38). In the geriatric Chinese population study, sensitivity was 77.6%, specificity was 74.7%, and overall accuracy was 75.8% (37).

The Arabic version's sensitivity (87.2%) falls between the general Chinese population (90.9%) and the geriatric Chinese population (77.6%) (37, 38). This intermediate performance may reflect differences in age distribution, as the Arabic study included adult patients across a broader age range. The Arabic version's specificity (75.6%) is higher than the general Chinese population (66.6%) but comparable to the geriatric Chinese population (74.7%) (37, 38). The overall accuracy of the Arabic version (81.8%) exceeds both Chinese adaptations (74.4 and 75.8%), (37, 38) suggesting favorable overall diagnostic performance.

These cross-cultural comparisons reveal interesting patterns. The original Korean study (17) achieved the highest specificity (89.8%), followed by the Arabic version (75.6%) and the Chinese versions (37, 38) (66.6%−74.7%). Conversely, the Chinese general population study (38) achieved the highest sensitivity (90.9%), followed by Arabic version (87.2%) and the original Korean (87.0%) (17) with the Chinese geriatric population (37) showing the lowest sensitivity (77.6%). These variations likely reflect complex interactions between cultural factors, linguistic characteristics, healthcare system differences, patient demographics, and methodological variations across studies.

The limited agreement between questionnaire-based and positional diagnoses for the affected ear and BPPV subtype, with a substantial proportion of cases classified as undetermined, suggests that the Arabic version may be more useful for screening purposes than for definitive subtype determination. This finding has important implications for the questionnaire's clinical application, indicating that while it can effectively identify patients who may benefit from further positional testing; it should not replace comprehensive clinical examination for treatment planning.

This finding suggests that the Arabic version may have lower performance for subtype determination compared to the original and Chinese versions (17, 37, 38). The lower internal consistency of the subtype determination questions (Cronbach's α = 0.532) further supports this interpretation. 26.8% of patients reported that both positional triggers provoked their vertigo and were therefore classified as undetermined. Possible explanations include challenges in patient comprehension of the specific symptom descriptors required for accurate subtype identification particularly in acute phase or cultural differences in spatial orientation and directional terminology.

The Arabic self-diagnosis BPPV questionnaire may serve as a useful screening tool in clinical settings where Arabic is the primary language of communication. Its high sensitivity (87.2%) and negative predictive value (83.8%) make it particularly valuable for ruling out BPPV when the questionnaire result is negative, potentially reducing unnecessary referrals for specialized positional testing. The moderate positive predictive value (80.4%) suggests that patients who screen positive should undergo confirmatory positional testing, consistent with the questionnaire's intended use as a screening rather than diagnostic instrument.

In primary care and general practice settings, where access to specialized vestibular assessment may be limited, the Arabic questionnaire could facilitate early identification of patients who would benefit from referral to specialists or otolaryngology clinics.

For research purposes, the Arabic questionnaire may be useful in epidemiological studies investigating BPPV prevalence, risk factors, and outcomes in Arabic-speaking populations. It may also serve as a screening instrument in clinical trials evaluating interventions for BPPV, though its limitations in subtype determination should be considered when designing such studies. The questionnaire could facilitate large-scale data collection in settings where comprehensive positional testing of all participants is not feasible.

This study has several methodological strengths that enhance confidence in the findings. First, the cross-cultural adaptation process rigorously followed established international guidelines (28), Second, the study employed comprehensive psychometric evaluation, including content validity, construct validity (both convergent and known-groups), reliability, and diagnostic accuracy assessment. Third, the use of the positional test as the reference standard is appropriate, as it represents the gold standard for BPPV diagnosis (1). Fourth, the study utilized *a priori* hypotheses for outcome evaluation, reducing the risk of *post-hoc* interpretation bias and enhancing the scientific rigor of the validation process.

However, several limitations should be acknowledged when interpreting the findings of this study. First, current study used relatively small convenience sampling method at a single center and did not include patients with horizontal canal involvement. This would limit the generalizability of the findings and the ability to test the diagnostic performance of questions 5 and 6. Second, the study did not assess responsiveness to change, which would be necessary to evaluate the questionnaire's utility in monitoring treatment outcomes. Third, the lower internal consistency of the subtype determination questions (Cronbach's α = 0.532) and the limited agreement for affected ear and subtype classification suggest that this component of the questionnaire may require further refinement or validation in the Arabic context.

Several directions for future research can build upon the findings of this study. For example, multicenter validation studies with larger sample sizes across different Arabic-speaking countries and regions are needed to establish the questionnaire's generalizability and to identify potential regional or dialectal variations in performance. Additionally, qualitative studies could be conducted to explore patient comprehension of questions 4–6 and cognitive interviewing to identify sources of difficulty or misinterpretation.

Moreover, longitudinal studies evaluating the questionnaire's responsiveness to change following treatment (e.g., canalith repositioning procedures) would provide important information about its utility in monitoring treatment outcomes. Research is needed to examine the questionnaire's utility in patient self-management, including self-administered canalith repositioning procedures for recurrent BPPV, would align with the original questionnaire's intended purpose (17) and could have important implications for patient empowerment and healthcare resource utilization. Additionally, comparative effectiveness studies evaluating clinical outcomes, patient satisfaction, and healthcare costs associated with questionnaire-based screening vs. usual care would provide valuable evidence for clinical decision-making and health policy.

## 5 Conclusion

This study provides initial evidence supporting the content validity, construct validity, and diagnostic performance of the Arabic self-diagnosis BPPV questionnaire for screening purposes in Arabic-speaking patients presenting with vertigo. The Arabic self-diagnosis BPPV questionnaire demonstrated strong content validity, acceptable construct validity, and good diagnostic performance for screening BPPV. While the questionnaire shows promise as a screening tool in clinical and research settings, its utility for definitive subtype determination requires further clarification.

## Data Availability

The raw data supporting the conclusions of this article will be made available by the authors, without undue reservation.

## References

[1] Bhattacharyya N Gubbels SP Schwartz SR Edlow JA El-Kashlan H Fife T . Clinical practice guideline: Benign paroxysmal positional vertigo (update). Otolaryngol Head Neck Surg. (2017) 156:S1–47. doi: 10.1177/0194599816689667 28248609

[2] von Brevern M Radtke A Lezius F Feldmann M Ziese T Lempert T . Epidemiology of benign paroxysmal positional vertigo: a population based study. J Neurol Neurosurg Psychiatry. (2007) 78:710–5. doi: 10.1136/jnnp.2006.100420 17135456 PMC2117684

[3] Kim JS Zee DS. Clinical practice. Benign paroxysmal positional vertigo. N Engl J Med. (2014) 370:1138–47. doi: 10.1056/NEJMcp1309481 24645946

[4] Parnes LS Agrawal SK Atlas J. Diagnosis and management of benign paroxysmal positional vertigo (BPPV). CMAJ. (2003) 169:681–93. 14517129 PMC202288

[5] Gopinath B McMahon CM Rochtchina E Mitchell P. Dizziness and vertigo in an older population: the blue mountains prospective cross-sectional study. Clin Otolaryngol. (2009) 34:552–6. doi: 10.1111/j.1749-4486.2009.02025.x 20070765

[6] Monzani D Casolari L Guidetti G Rigatelli M. Psychological distress and disability in patients with vertigo. J Psychosom Res. (2001) 50:319–23. doi: 10.1016/S0022-3999(01)00208-2 11438113

[7] Yardley L Owen N Nazareth I Luxon L. Prevalence and presentation of dizziness in a general practice community sample of working age people. Br J Gen Pract. (1998) 48:1131–5. 9667086 PMC1410052

[8] Benecke H Agus S Kuessner D Goodall G Strupp M. The burden and impact of vertigo: findings from the REVERT patient registry. Front Neurol. (2013) 4:136. doi: 10.3389/fneur.2013.00136 24106487 PMC3788351

[9] Lopez-Escamez JA Gamiz MJ Fernandez-Perez A Gomez-Fiñana M. Long-term outcome and health-related quality of life in benign paroxysmal positional vertigo. Eur Arch Otorhinolaryngol. (2004) 262:507–11. doi: 10.1007/s00405-004-0841-x 15942805

[10] Hanley K O'Dowd T Considine N. A systematic review of vertigo in primary care. Br J Gen Pract. (2001) 51:666–71. 11510399 PMC1314080

[11] Neuhauser HK. Lempert T. Vertigo: eEpidemiologic aspects. Semin Neurol. (2009) 29:473–81. doi: 10.1055/s-0029-1241043 19834858

[12] Alharbi AA Alshammari ME Albalwi AA Ramadan MM Alsharif DS Hafiz AE. Dizziness in Saudi Arabia: an epidemiologic study. Front Neurol. (2023) 14:1040231. doi: 10.3389/fneur.2023.1040231 37090980 PMC10117996

[13] Shami I Al Sanosi A. Causes of vertigo in Saudi patients seen at tertiary teaching hospital. J Taibah Univ Med Sci. (2011) 6:26–32. doi: 10.1016/S1658-3612(11)70153-6

[14] Nunez RA Cass SP Furman JM. Short- and long-term outcomes of canalith repositioning for benign paroxysmal positional vertigo. Otolaryngol Head Neck Surg. (2000) 122:647–52. doi: 10.1067/mhn.2000.105185 10793340

[15] Sakaida M Takeuchi K Ishinaga H Majima Y. Long-term outcome of benign paroxysmal positional vertigo. Neurology. (2003) 60:1532–34. doi: 10.1212/01.WNL.0000061477.03862.4D 12743247

[16] Kim HJ Kim JS. The patterns of recurrences in idiopathic benign paroxysmal positional vertigo and self-treatment evaluation. Front Neurol. (2017) 8:690. doi: 10.3389/fneur.2017.00690 29326650 PMC5736533

[17] Kim HJ Song JM Zhong L Yang X Kim JS. Questionnaire-based diagnosis of benign paroxysmal positional vertigo. Neurology. (2020) 94:e942–9. doi: 10.1212/WNL.0000000000008876 31888973

[18] Jacobson GP Newman CW. The development of the dizziness handicap inventory. Arch Otolaryngol Head Neck Surg. (1990) 116:424–7. doi: 10.1001/archotol.1990.01870040046011 2317323

[19] Yardley L Masson E Verschuur C Haacke N Luxon L. Symptoms, anxiety and handicap in dizzy patients: development of the Vertigo Symptom Scale. J Psychosom Res. (1992) 36:731–41. doi: 10.1016/0022-3999(92)90131-K 1432863

[20] Zmnako SSF Chalabi YI. Cross-cultural adaptation, reliability, and validity of the Vertigo symptom scale–short form in the central Kurdish dialect. Health Qual Life Outcomes. (2019) 17:125. doi: 10.1186/s12955-019-1168-z 31315639 PMC6637568

[21] Hall CD Herdman SJ Whitney SL Cass SP Clendaniel RA Fife TD . Vestibular rehabilitation for peripheral vestibular hypofunction: an evidence-based clinical practice guideline: from the American Physical Therapy Association Neurology Section. J Neurol Phys Ther. (2016) 40:124–55. 26913496 10.1097/NPT.0000000000000120 PMC4795094

[22] Swartz R Longwell P. Treatment of vertigo. Am Fam Physician. (2005) 71:1115–22. 15791890

[23] Nada EH Galhom DH Elsayed AA Abdalla AA Hegazy MA Saleh MS . Validity and reliability of the Arabic version of vestibular rehabilitation benefit questionnaire. Egypt J Otolaryngol. (2025) 41:10. doi: 10.1186/s43163-025-00836-0

[24] Whitney SL Wrisley DM Brown KE Furman JM. Is perception of handicap related to functional performance in persons with vestibular dysfunction? Otol Neurotol. (2004) 25:139–43. doi: 10.1097/00129492-200403000-00010 15021773

[25] World Bank. Population, Total—Arab World. (2023). Available online at: https://data.worldbank.org/indicator/SP.POP.TOTL?locations=1A (accessed January 13, 2026).

[26] Coskun Benlidayi I Gupta L. Translation and cross-cultural adaptation: a critical step in multi-national survey studies. J Korean Med Sci. (2024) 39:e336. doi: 10.3346/jkms.2024.39.e336 39716865 PMC11666326

[27] Epstein J Santo RM Guillemin F. A review of guidelines for cross-cultural adaptation of questionnaires could not bring out a consensus. J Clin Epidemiol. (2015) 68:435–41. doi: 10.1016/j.jclinepi.2014.11.021 25698408

[28] Beaton DE Bombardier C Guillemin F Ferraz MB Patrick DL. Guidelines for the process of cross-cultural adaptation of self-report measures. Spine. (2000) 25:3186–91. doi: 10.1097/00007632-200012150-00014 11124735

[29] Anthoine E Moret L Regnault A Caret V Hardouin JB. Sample size used to validate a scale: a review of publications on newly-developed patient reported outcomes measures. Health Qual Life Outcomes. (2014) 12:176. doi: 10.1186/s12955-014-0176-2 25492701 PMC4275948

[30] Bujang MA Adnan TH. Requirements for minimum sample size for sensitivity and specificity analysis. J Clin Diagn Res. (2016) 10:YE01–6. doi: 10.7860/JCDR/2016/18129.8744 PMC5121784 27891446

[31] Terwee CB Bot SDM de Boer MR van der Windt DAWM Knol DL Dekker J . Quality criteria were proposed for measurement properties of health status questionnaires. J Clin Epidemiol. (2007) 60:34–42. doi: 10.1016/j.jclinepi.2006.03.012 17161752

[32] Zamanzadeh V Ghahramanian A Rassouli M Abbaszadeh A Alavi-Majd H Nikanfar AR. Design and implementation content validity study: development of an instrument for measuring patient-centered communication. J Caring Sci. (2015) 4:165–78. doi: 10.15171/jcs.2015.017 26161370 PMC4484991

[33] Polit DF Beck CT Owen SV. Is the CVI an acceptable indicator of content validity? Appraisal and recommendations. Res Nurs Health. (2007) 30:459–67. doi: 10.1002/nur.20199 17654487

[34] Viera AJ Garrett JM. Understanding interobserver agreement: the kappa statistic. Fam Med. (2005) 37:360–63. 15883903

[35] Çorbacioglu SK Aksel G. Receiver operating characteristic curve analysis in diagnostic accuracy studies: a guide to interpreting the area under the curve value. Turk J Emerg Med. (2023) 23:195–98. 38024184 10.4103/tjem.tjem_182_23 PMC10664195

[36] Cronbach LJ. Coefficient alpha and the internal structure of tests. Psychometrika. (1951) 16:297–34. doi: 10.1007/BF02310555

[37] Wan Y Li Y Sun J. The reliability of a subtype-determining questionnaire in efficient benign paroxysmal positional vertigo diagnosis in geriatrics. Front Aging Neurosci. (2023) 15:1209342. doi: 10.3389/fnagi.2023.1209342 37409007 PMC10318130

[38] Wan Y Li Y Zhou L Li H Kong W Lyu Y. Significance of subtype-determining questionnaire in efficient diagnosis and treatment of BPPV. Acta Oto-laryngol. (2023) 143:106–12. doi: 10.1080/00016489.2023.2166987 36662151

